# The generative AI ethics landscape as seen by Chinese middle school students

**DOI:** 10.3389/fpsyg.2026.1777855

**Published:** 2026-03-16

**Authors:** Yanyan Zhang, Xin Wan, Suping Yi, Yefeng Lu

**Affiliations:** 1 College of Education Science and Technology, Nanjing University of Posts and Telecommunications, Nanjing, China; 2 Danyang Xinqiao Junior High School, Zhenjiang, China; 3 School of Education Science, Jiangsu Second Normal University, Nanjing, China; 4 School of Teacher Education, Huzhou University, Huzhou, China

**Keywords:** AI ethical principles, AI ethics, AI ethics education, generative artificial intelligence, middle school students

## Abstract

With the rapid integration of Generative AI in education, understanding students' ethical perspectives is crucial for effective AI ethics education. Five hundred and ninety four middle school students' agreement levels on five AI ethical principles (beneficence, non-maleficence, justice, autonomy, explicability) adapted from previous research, and the rationales underlying their choices were investigated using a questionnaire. Results showed that students expressed the highest agreement with “beneficence” and “autonomy,” though overall responses leaned toward neutrality. Independent AI use and family discussions predicted higher agreement; urban-rural differences were non-significant. Qualitative analysis identified themes in students' ethical reasoning. These findings offer evidence-based guidance for adolescent AI ethics education.

## 1 Introduction

Currently, generative artificial intelligence (generative AI) is undergoing rapid expansion worldwide. Except for ChatGPT, a variety of large language model (LLM)-based applications such as DeepSeek, ERNIE Bot, and Doubao has emerged within China. Numerous AI-driven tools are now making their way into K−12 education, leveraging their advanced content-generation capabilities to offer students unparalleled support and convenience (Zhang et al., 2025). From language translation and writing assistance to generating problem-solving strategies, planning trips, and even engaging in social interactions, it can be said that generative AI has become embedded in learning and daily life (Gouseti et al., 2025).

According to the Report on Adolescents' Internet Usage (2024), 45.1% of adolescents have used AI products (Tencent Horizon, 2024). Yet alongside these benefits, the ethical risks and challenges associated with generative AI are increasingly coming to the forefront. Students turning to AI to complete assignments has ignited debates over academic integrity. Algorithms that gather excessive personal data have raised privacy concerns. and implicit biases (such as gender and regional stereotypes) present in generated content may mislead adolescents whose values are still developing (Miao F., 2024). These issues have positioned generative AI ethics as a central topic in education systems worldwide.

In response to the ethical challenges posed by artificial intelligence, China has taken measures at the policy level. On September 25, 2021, the National Governance Committee for the New Generation Artificial Intelligence released the “Ethical Norms for the New

Generation Artificial Intelligence,” which identifies the enhancement of ethical literacy as one of the six fundamental ethical requirements for AI. The document explicitly calls upon AI users to actively learn about and disseminate AI ethics knowledge, as well as to initiate or participate in discussions on AI-related ethical issues (Ministry of Science Technology of the People's Republic of China, 2021). This provides a solid ethical foundation for fostering harmonious human-machine relationships, protecting public interests, and promoting the healthy development of the AI industry. However, before the widespread adoption of generative AI, factors such as high costs and maintenance requirements limited its integration into education, and consequently, AI ethics did not receive adequate attention from educators and researchers (Memarian and Doleck, 2023).

To advance the integration and development of AI education in primary and secondary schools and to improve students' AI literacy, the Ministry of Education of the People's Republic of China. (2024) issued a notice in 2024 to explore implementation pathways for AI education in these schools and strengthen AI education. Consequently, integrating AI ethics into teaching has become an urgent task for teachers, who must guide students in using AI technologies appropriately within the curriculum (Yang et al., 2025).

Although research on AI ethics in education has expanded in response to policy initiatives and teaching needs, there remains limited research examining AI ethics from the perspective of students. Furthermore, existing technology ethics education and AI ethics education in China are predominantly focused at the university (Li et al., 2024), overlooking the distinct cognitive characteristics of younger learners such as middle school students. Without targeted studies on this specific group, teachers face challenges in assessing the relevance and suitability of AI ethics content for their students. This can lead to a disconnect between ethics instruction and students' actual experiences, missing opportunities to address practical dilemmas in the application of AI ethics. In light of this, our study focuses on middle school students, aiming to empirically map the landscape of their perceptions of generative AI ethics. The findings are expected to provide an evidence-based foundation for designing and implementing age-appropriate generative AI ethics education for this population.

## 2 Literature review

### 2.1 AI ethics framework

AI ethics refers to the principles and rules that need to be followed during the development, application, and governance of artificial intelligence (Chen and Zhu, 2024). After the necessity of AI ethics gained widespread acceptance, society began to explore ethical guidelines for AI to help navigate the competing human interests implicated by AI technologies (Jobin et al., 2019) and form the theoretical foundation of AI ethics education. Governments and international organizations have progressively established ethical principles and guidelines for AI. For instance, the Executive Office of the President, and the Office of Management and Budget (2020) issued the “Guidance for Regulation of Artificial Intelligence Applications,” outlining 10 AI ethical principles including public trust in AI, public participation, and scientific integrity and information quality in 2020. In 2021, UNESCO (2021) released the “Recommendation on the Ethics of Artificial Intelligence,” which includes 10 principles such as proportionality and do no harm, fairness and non-discrimination, and safety and security. Similarly, the European Commission's High-Level Expert Group on Artificial Intelligence (2019) published “Ethics Guidelines for Trustworthy AI,” structured around respect for human autonomy, prevention of harm, and fairness and explicability.

Numerous scholars have also contributed significantly to developing AI ethics frameworks. Early research tended to concentrate on a limited set of core dimensions. For instance, Inuwa-Dutse (2023) proposed the FATE framework, which is built on fairness, accountability, and transparency. The final letter “E” in FATE stands for “Ethics,” serving as an umbrella term summarizing the first three dimensions (Memarian and Doleck, 2023). Likewise, Cath et al. (2018) and Daly et al. (2019) proposed frameworks organized around three ethical principles. As research progressed, some scholars attempted to develop more comprehensive ethical systems. Nguyen et al. (2022) suggested seven ethical principles for educational contexts; Jobin et al. (2019) identified 11 principles through a systematic literature review; and Schiff et al. (2021) proposed an extensive framework including 25 dimensions, covering a wide range of aspects such as social responsibility, human rights, fairness, trust, and cultural sensitivity. Corrêa et al. (2023) extracted 17 categories of AI principles in a meta-analysis of 200 AI policy documents. However, this expansion of dimensions has also led to conceptual overlaps and systemic redundancy, to some extent hindering the dissemination and application of ethical guidelines in educational practice (Schiff, 2022).

To address this fragmentation, Floridi and Cowls (2022) synthesized 47 existing ethical principles into an integrated framework of five-dimensions: beneficence, non-maleficence, justice, autonomy, and explicability. Adams et al. (2023) analyzed documents published by four international institutions to summarize and explain the commonalities across published ethical guidelines. Chinese scholars Chen and Zhu (2024) performed a coding analysis of six policy documents from governments and international organizations, proposing five major AI ethical principles. These efforts aim to create logically distinct and structurally clear frameworks, laying a foundation for the unification and effective dissemination of AI ethics. Research on AI ethical frameworks demonstrates a trend from diversity and fragmentation toward systematic integration.

Current research on AI ethics frameworks predominantly employs qualitative research methods. In China, for example, studies often focus on philosophical and conceptual analysis, yet a unified AI ethics framework has not been established. Or some researchers constructed AI ethics framework through literature review (Adams et al., 2023). While theoretical research is undoubtedly important, the construction and discussion of ethical frameworks from the perspectives of experts or authorities lack the experiential wisdom and input from student perspectives, particularly from adolescents. This may create a gap between theory and practice, leaving AI ethics education overly generalized or simplified. Adopting an evidence-based approach to study AI ethics with adolescents presents a valuable opportunity to expand and deepen the boundaries of AI ethics research.

### 2.2 Student perceptions of AI ethics

As ethical concerns surrounding artificial intelligence in education continue to grow, many researchers have examined students' awareness and understanding of AI ethics. Most studies focused on topics most closely related to students. For instance, Lund et al. (2025) investigated 277 university students to examine their perceptions and awareness toward academic misconduct. The findings indicated that while students showed a strong awareness and concern about using AI for writing papers, there was considerable difference across demographic groups regarding their understanding of the serious consequences associated with academic misconduct. A survey of da Silva et al. (2024) focused on undergraduates' perceptions of AI tools in their academic work. Four primary areas of concern: the authenticity and accuracy of information, plagiarism and ethics in the academic use of AI, the diminution of critical thinking or learning skills, and dependence on AI were identified. Yu (2023) explored whether ChatGPT should be banned by academia by investigating 200 university students who used AI-assisted writing tools. Eighty percentage of students concerned that overuse or dependence on AI could diminish independent thought. In a study by Lee and Maeng (2023), 30 South Korean high school students were investigated regarding their perceptions of AI systems. The research also uncovered students' ethical concerns regarding plagiarism and copyright issues in chatbot usage.

Attitude toward AI and AI literacy are concept closely related to and often overlapping with AI ethics. Therefore, many scholars have conducted surveys on attitudes toward artificial intelligence and AI literacy, treating AI ethics as only a minor aspect of these two concepts. For instance, Weichert et al. (2025) used an attitude toward AI scale to investigate 117 university students, aiming to understand their perspectives on general attitudes toward AI, AI ethics, and AI regulation and policy. Findings from the AI ethics section revealed considerable variation in students' views on ethical issues related to AI. Students showed the highest level of concern about current issues such as deepfakes, misinformation, and data privacy, while also showing greater concerns about AI's impact on human emotions and behavior, as well as job loss due to increased automation in the future context.

Further research has analyzed how students' perceptions of AI tools vary based on background factors. Basch et al. (2025) found that university students who use AI frequently show higher levels of trust in it. They were more likely to believe that AI facilitates learning and enhances writing, critical thinking, and communication skills. In contrast, infrequent users tended to view AI use in the classroom as a form of cheating, expressed distrust in AI security, and emphasized the need for stricter regulation. Tlili et al. (2023) also examined differences among students with different educational and cultural backgrounds. Their research revealed that Western participants were more concerned about plagiarism and academic misconduct, while Asian participants placed greater emphasis on privacy risks. Global data indicated that 55% of students used AI tools to enhance their writing and problem-solving abilities, yet 70% of respondents believed that such use led to a decline in critical thinking skills. These findings demonstrated differences in how students perceive the AI ethics.

A limited number of previous researches recognized that existing research on AI ethics predominantly focused on topics such as academic integrity and plagiarism, resulting in a fragmented understanding of students' ethical perceptions. Consequently, drawing on the AI ethics framework proposed by Floridi and Cowls (2022), (Colón-Aguirre and Bright, 2025) employed qualitative research methods to explore undergraduate students' perceptions of AI tools in completing course assignments. Similarly, Yang et al. (2025) investigated the extent to which university students identify with five key AI ethical principles and their underlying rationale. Their findings illustrated that students' understanding of ethical issues in AI use has expanded beyond plagiarism. However, notable gaps remain in their comprehension of the true nature and broader impacts of AI.

The above results suggest that ethical frameworks should align with students‘ perceptions of responsible AI use, and that customized educational strategies need to be developed based on students' cognitive levels to promote balanced ethical reflection (Ko and Song, 2025). While these researches are valuable, the literature reveals two major limitations. First, there is a narrow focus in terms of research participants. Most studies focused on university students with significantly less attention given to primary and secondary school students. Second, the research outcomes remain partial. Investigations are largely confined to students‘ understanding of specific issues like academic misconduct, lacking in-depth exploration of systematic ethical principles. This results in a fragmented and superficial understanding of students' AI ethical cognition.

### 2.3 Current practices in AI ethics education

AI ethics is extremely complex, AI ethics education correspondingly must be similarly complex. In particular, scholars emphasize that practitioners should not only understand AI ethical principles or issues but also how to integrate ethics into the education. Fortunately, universities in many countries have already initiated AI ethics education, with numerous institutions launching responsible AI programs (Wiese et al., 2025). For instance, the Massachusetts Institute of Technology has implemented a university-wide Responsible AI Initiative (MIT, 2024), while Stanford University has long supported the development of its Institute for Human-Centered Artificial Intelligence.

According to studies by Garrett et al. (2020), Raji et al. (2021), and Javed et al. (2022), standalone AI ethics courses are one of the main forms of AI ethics education in the United States, North America, and globally. These courses typically address socio-ethical issues, philosophical ethical theories, ethical and legal norms, and the development of AI technology, using professional publications, literary and cinematic works, and ethical cases as instructional materials. However, the separation of ethics from technology in these standalone courses often limits the achievement of AI ethics education objectives.

Therefore, embedding ethics modules into existing technology courses represents another important form of AI ethics education. This form is widely advocated because it overcomes the limitations of the standalone courses and is less constrained by factors such as course hour requirements. By transcending the limitations of ethics education delivered by standalone courses, it enables students to engage with ethics content related to specific technologies while learning those technologies. This includes theoretical ethics modules and applied ethics modules. Theoretical ethics modules provide instruction on ethical knowledge that matches the technology topic, such as discussing bias and anonymization in data science or machine learning. Applied ethics modules integrate ethical elements into practical technology exercises, helping students recognize how their technical choices affect others and society (Bai and Yu, 2025).

As a leading and trending technology, AI education is viewed as crucial for adapting to technological trends and building a talent pipeline for national competitiveness. It carries the important mission of stimulating students' innovative potential and enhancing their practical abilities. Consequently, under the influence of a prevailing technological optimism in society, AI education emphasizes the innovative potential of AI, while ethical considerations have not received attention comparable to that given to technical knowledge and skills. AI ethics education is often fall by the wayside—— “if time allows” ——in the curriculum (Garrett et al., 2020). The practice of AI ethics education remains at an early stage, facing significant limitations such as a focus on higher education, an emphasis on academic integrity in content, and a lack of systematic teaching practices and assessments (Wiese et al., 2025). AI ethics education cannot adopt a one-size-fits-all approach, as students at different ages have distinct cognitive characteristics. There is an urgent need to advocate for dedicated AI ethics education, particularly for minors.

## 3 Research questions

The above literature reveals diverse perspectives on AI ethics and highlights a general lack of awareness and inconsistent application of ethical principles (Kamali et al., 2024). What's more, we still lack sufficient information on how students perceive these AI tools from an ethical perspective (Colón-Aguirre and Bright, 2025), especially for minors. Therefore, in the context of the Ministry of Education of the People's Republic of China strengthening AI education in primary and secondary schools, the research objective of this study is to examine middle school students' perceptions of AI ethics. To achieve this research objective, the study addresses three research questions. First, what is the level of agreement among middle school students with AI ethical principles? Second, are there differences in the level of agreement among different students? Third, what are the rationales for students' agreement or disagreement with AI ethical principles? The findings are expected to help educators further refine the requirements for AI education, provide support for AI teaching, design better programs and interventions to enhance students' understanding of AI ethics.

## 4 Method

### 4.1 Participants

This study involved students in grades 7 through 9 from two public middle schools in Zhenjiang City, Zhejiang Province—one located in an urban area and the other in a township. Teachers at both schools have incorporated generative AI into their teaching and all students are aware of what generative AI is. A random cluster sampling method was employed within two schools to select participants. Specifically, three intact classes were randomly selected from each of the three grades (7, 8, and 9). All students in these selected classes were included in the study. This approach effectively avoided selection bias at the individual level and maintained the natural educational setting, thereby facilitating the collection of authentic data.

A total of 625 middle school students were recruited for the survey. After data collection, rigorous data cleaning was conducted. Thirty one invalid questionnaires due to incomplete responses were excluded. Ultimately, 594 valid questionnaires were retained, with an effective response rate of 95.04%. Participants' demographic information are summarized in Table 1. All the participants were in the age range of 12-16 years (*M* = 13.76, *SD* = 0.936). Among the valid samples, males accounted for 52.2% (*N* = 310) and females for 47.8% (*N* = 284). The grade distribution was as follows: 170 seventh-grade students (28.6%), 221 eighth-grade students (37.2%), and 203 ninth-grade students (34.2%). In terms of geographical distribution, 43.1% of the students were from urban areas (*N* = 256) and 56.9% were from township areas (*N* = 338). The sample demonstrated balanced demographic representation, providing a solid basis for subsequent analysis.

**Table 1:** The participants' demographic information.

**Items**	**Categories**	**N (%)**
Gender	Male	310 (52.2%)
	Female	284 (47.8%)
Grade	1	170 (28.6%)
	2	221 (37.2%)
	3	203 (34.2%)
Location	Urban	256 (43.1%)
	Township	338 (56.9%)
Age	12	48 (8.1%)
	13	197 (33.2%)
	14	210 (35.4%)
	15	130 (21.9%)
	16	9 (1.5%)

### 4.2 Research instrument

This study employed a questionnaire adapted from the work of Yang et al. (2025). The questionnaire consists of two parts. The first part includes students' basic information (e.g., gender, age, grade, and school location). The second part addresses student agreement with AI ethical principles and their rationales.

The AI ethics framework guiding this study was the unified model proposed by Floridi and Cowls (2022), which comprises five core principles: beneficence, non-maleficence, justice, autonomy, and explicability. Beneficence means AI technologies which are beneficial to humanity while preserving human dignity and sustaining the goodness of humanities. Non-maleficence is defined as the use of artificial intelligence technologies should not harm humanity, with particular attention to protecting people's privacy from being violated. While justice is defined as the promotion of prosperity and eliminating all forms of bias in AI applications. Autonomy refers to AI should enhance human decision-making rather than deprive individuals of their power to make independent decisions. humans should retain ultimate control over when and how to use AI, where the delegation of tasks to AI technologies should be overridable at any point by human agents. Explicability is presented as the related knowledge should remain transparent to prevent human beings from being dominated by AI technology.

This framework was chosen because it is widely recognized and applied by other scholars, and the five core principles are mutually exclusive, easy for students to understand, and unlikely to cause ambiguity (Yang et al., 2025). An example of the question is as follows. Non-maleficence: Generative AI could violate people's privacy rights. Participants were asked to rate their level of agreement with each statement regarding AI ethics, ranging from 1 (strongly disagree) to 5 (strongly agree). Subsequently, students were required to provide rationales for their choices in an open-ended question.

For each ethical principle, only two questions were designed: one single-choice question and one open-ended question. This is because the study is a preliminary exploration based on the perspective of middle school students, with a focus not on developing a complex and rigorously validated scale. In this study, students‘ agreement with five ethical principles; and the initial rationales expressed by students in open-ended questions, to some extent, could reflect students' perceptions of AI ethics. We acknowledge that research findings reflect preliminary attitudinal tendencies rather than stable or comprehensive ethical judgments.

The questionnaire underwent translation between English and Chinese. First, a PhD student majored in technology education who is good at English translated the English scale into Chinese. Subsequently, another bilingual researcher who had not been exposed to the original scale back-translated the Chinese version into English. The research team compared the back-translated version with the original, discussed and revised items with semantic discrepancies, and repeated the above translation process until semantic consistency was achieved between the Chinese and English versions.

We invited one middle school teacher and 3 students to read these items to ensure each statement is easy to understand for middle school students. Content validity was assessed by four experts familiar with scale development and the artificial intelligence applications. They rated each item's relevance to the target construct (1 = not relevant, 4 = highly relevant). All items achieved an item-level content validity index (I-CVI) of 1.00, and the scale-level index (S-CVI) was 1.00, indicating excellent content validity (Polit and Beck, 2006). We also conducted pilot-test item–total correlations analysis (*N* = 120). Results show that 5 items correlations range from 0.408 to 0.566, indicating that each item effectively reflects its intended construct (DeVellis and Thorpe, 2021). The overall Cronbach's Alpha for the questionnaire was 0.738. Notably, the alpha coefficient would decrease if any single item were removed. The above two confirming that all five items are valid and contribute positively to the scale's reliability and should be remained.

### 4.3 Data collection

Before the formal survey, the research team obtained approval from the administrative departments of the participating schools. Active informed consent was obtained from both parents and students. The consent forms clearly outlined the research purpose, procedures, data confidentiality measures, and the voluntary nature of participation, emphasizing that students could withdraw from the study at any time without any consequences.

Given that middle school students have limited access to smartphones, data were collected using paper-based questionnaires. Before students filling out the questionnaire, teachers and research team members jointly introduced the meanings of five AI ethical principles to ensure students understood their meanings. The questionnaires were completed during class time, which provided a standardized setting and allowed for timely clarification of any questions raised by students. On average, it took students approximately 5 min to complete the questionnaire. After collection, responses were manually entered into an Excel spreadsheet by the research team.

### 4.4 Data analysis

The study primarily utilized SPSS 27 for data analysis. First, after completing data entry, all variables were cleaned and verified to ensure data integrity. Descriptive analysis was conducted on the sample's demographic variables (such as school location) as well as scores on the five AI ethical principles. Second, to account for the hierarchical structure of the data (students nested within classes), a linear mixed-effects model analysis was performed.

Thematic coding analysis was applied to the open-ended responses. A single response was treated as the minimal codable textual unit. Initially, an inductive approach was used to identify emerging themes from the students' responses after one researcher reading through and revisit the data. These themes were then refined into a structured codebook. To ensure the reliability of the coding process, two independent researchers coded 20% of the responses blindly. The inter-rater reliability was calculated using Cohen's Kappa coefficient, resulting in a value of 0.93, indicating strong agreement (Landis and Koch, 1977). All coding disagreements were resolved through discussions. For example, “it has both good and bad aspects, I can't explain it clearly.” Coder A categorized it as a contradiction and trade-off. Coder B categorized it as a cognitive limitation. After discussion, it was determined to categorize it under cognitive limitation, because the phrase “both good and bad” in this response represents only a vague intuition rather than a specific analysis of pros and cons. The phrase “I can't explain it clearly” reflects the student's insufficient depth of understanding.

## 5 Results

### 5.1 Descriptive analysis of student's agreement of AI ethical principles

The mean value and the SD value of the five principles were shown in the Table 2. The higher the values, the higher the student's level of agreement of ethical principles. It can be seen that mean values of beneficence and autonomy fall between 3 = neutral and 4 = agree, and is closer to 3. It indicates that students do not totally agree with the statement referring to beneficence and autonomy. Furthermore, compared to other principles' mean value, the mean value of the non-maleficence, justice and explicability are lower than 3, indicating that students are especially disagree with statement of these three AI ethical principles.

**Table 2:** Descriptive statistics of the five principles and the whole questionnaire.

**Variables**	**M**	**SD**
Beneficence	3.12	0.885
Non-maleficence	2.92	0.939
Justice	2.93	0.840
Autonomy	3.10	0.923
Explicability	2.96	0.732

### 5.2 Differences analysis of student's agreement of AI ethical principles

To examine the differences in agreement levels across the five AI ethical principles while accounting for the nested data structure, a linear mixed-effects model was conducted. The model included the ethical principle as a fixed effect, with random intercepts for student and class to account for the nested data structure. Results in Table 3 and Table 4 revealed significant differences among student's agreement level on the five ethical principles (F _(4, 2371.97)_ = 10.13, *p* < 0.001). *Post-hoc* pairwise comparisons with Bonferroni adjustment indicated that students expressed the highest levels of agreement with Beneficence (*M* = 3.12) and Autonomy (*M* = 3.10), both of which were significantly higher than Non-maleficence (*M* = 2.92), Justice (*M* = 2.93), and Explicability (*M* = 2.96). No significant differences were found among the latter three principles.

**Table 3:** Comparison of students' agreement levels across five AI ethical principles.

**Variables**	***M***	**S.E**.	**95%CI**
Beneficence	3.12	0.043	[3.029, 3.203]
Autonomy	3.10	0.043	[2.830, 3.005]
Explicability	2.96	0.043	[2.843, 3.018]
Justice	2.93	0.043	[3.005, 3.180]
Non-maleficence	2.92	0.043	[2.870, 3.045]

**Table 4:** Pairwise Comparisons of five AI ethical principles.

***Comparison (I-J)***	***Mean Difference***	***S.E*.**	***p-value***
1 vs. 2	0.199	0.042	< 0.001
1 vs. 3	0.185	0.042	< 0.001
1 vs. 4	0.024	0.042	1.000
1 vs. 5	0.158	0.042	0.002
2 vs. 3	−0.013	0.042	1.000
2 vs. 4	−0.175	0.042	< 0.001
2 vs. 5	−0.040	0.042	1.000
3 vs. 4	−0.162	0.042	0.001
3 vs. 5	−0.027	0.042	1.000
4 vs. 5	0.135	0.042	0.013

1 = Beneficence, 2 = Autonomy, 3 = Explicability, 4 = Justice, 5 = Non-maleficence.

A linear mixed-effects model was conducted to examine whether students' agreement differed by their school location, using experience and family discussion. The model included three variables as a fixed effect and a random intercept for classID to account for the nested structure of the data. Table 5 showed the results. Students' school location information was collected via a choice item including two options: urban and township. The results indicate that students' agreement did not significantly differ based on their location (*p* = 0.472). Students' information of prior using experience with generative AI was collected via a single-choice question (yes/no). “Yes” means students used generative AI independently while “no” means students didn't use generative AI independently. Prior usage experience was a significant predictor (*b* = −0.154, *SE* = 0.062, *p* = 0.013) Students with AI using experience scored significantly higher than those who had not. Similarly, A single-choice question, including two choices (yes/no), was designed to investigate whether students had discussed AI-related topics with their parents. Family discussion experience was a significant predictor (*b* = −0.161, *SE* = 0.054, *p* = 0.003). Students who discussed AI with parents demonstrated higher agreement with AI ethical principles.

**Table 5:** Results of Mixed Linear Model by location, using experience and family discussion.

**Parameter**	**Estimate**	**S.E**.	***t-*statistics**	***p*-value**
location	−0.051	0.069	−0.738	0.472
using	−0.154	0.062	−2.501	0.013
family	−0.161	0.054	−3.016	0.003

### 5.3 The rationale behind students' agreement with AI ethics

During the data preprocessing stage, we re-categorized students' responses to analyze their stance on AI ethics and their rationales for their choices. The categorization was conducted as follows: “Strongly Agree” and “Agree” were merged into an “Agree” category, while “Strongly Disagree” and “Disagree” were merged into a “Disagree” category. “Neutral” was analyzed independently. This grouping decision was because middle school students are at a critical stage of development in moral judgment and abstract thinking. Their “neutral” responses often do not indicate an absence of position, but rather may reflect their unique cognitive characteristics. Our analysis of qualitative open-ended data further reveals that “neutral” responses are driven by diverse motivations. Therefore, to form clearer and more unified categories, the responses were ultimately divided into three groups: “Agree,” “Disagree” and “Neutral.” The detailed rationales stated by students are compiled in Table 6 (rationales for agreement), Table 7 (rationales for disagreement) and Table 8 (rationales for “Neutral” responses).

**Table 6:** Rationales behind students' agreement with AI ethical principles.

**AI ethical principles**	**Themes**	**Frequencies**
Beneficence	Academic integrity	105
	Fraud using AI	66
	Inaccurate information	32
Non-maleficence	Data privacy	112
Justice	Job displacement	68
	Inaccessibility	47
Autonomy	Overreliance on AI	92
	Skill degradation	64
Explicability	Ignore the process	44
	Unknown sources	23

**Table 7:** Rationales behind students' disagreement with AI ethical principles.

**AI ethical principles**	**Themes**	**Frequencies**
Beneficence	Human responsibility	56
Non-maleficence	Resolved	43
	Avoidable	38
	Overstatement	22
Justice	Support disadvantaged	64
	Benefit all	48
Autonomy	Cognitive empowerment	67
Explicability	Result-oriented	46
	Can be solved	19

**Table 8:** The rationales of “Neutral” responses.

**AI ethical principles**	**Themes**	**Frequencies**
Beneficence Non-maleficence Justice Autonomy Explicability	Cognitive limitations	89
	Context-dependence	67
	Contradiction and trade-offs	41
	Passive avoidance	15

As shown in Table 6, 105 students believed the biggest ethical issue with generative AI is that it leads to academic integrity problems. In addition, 66 students believed that generative AI technology provides more opportunities for deepfake fraud. Furthermore, 32 students pointed out that the content generated by generative AI has accuracy issues. Regarding the ethical principle of “non-maleficence,” 112 students noting that generative AI violates users data privacy. For the ethical principle of “justice,” students' rationales mainly involved two aspects: job displacement and inaccessibility. Sixty eight students believed that generative AI would replace some people's jobs, and 47 students thought that those who cannot apply or do not have access to generative AI would be at a disadvantage. Regarding the principle of “autonomy,” 92 students stated that people would become overly reliant on generative AI, while 64 students suggested that the use of AI applications might diminish our skills. For the principle of “explicability,” 44 students believed that the biggest ethical issue with generative AI is that generative AI does not provide users with the process, only the results. Twenty three students pointed out that the information provided by AI lacks sources.

From Table 7, for the ethical principle of “beneficence,” 56 students pointed out that AI ethical issues are caused by human. For “non-maleficence,” 43 students thought this issue has already been solved. Thirty eight students believed that this issue can be avoid while 22 students said this issue is overstatement. Regarding “justice,” 64 students thought AI can be used to support disadvantaged people. In addition, 48 students demonstrated that AI is inherently designed for human and is undoubtedly beneficial to human. For the ethical principle of “autonomy,” 67 students indicated that AI can empower cognition. Regarding “explicability,” 46 students with a results-oriented mindset. Another group of students believed that this ethical issue could be resolved as long as we persistently question and explore it.

From Table 8, we can see that students' rationales of “Neutral” can be categorized into four primary types. First, cognitive limitations. Students stated that “I haven't thought about this before” or “I haven't used AI enough to have an opinion on this,” indicating that their neutrality was a function of knowledge gaps or genuine uncertainty rather than a formed opinion. Second, context-dependence. Forty seven students demonstrated that their neutrality was an active, deliberative position, as they argued that “It all depends on how you use it” or “vary from case to case.” Third, contradiction and trade-offs. Thirty one respondents expressed genuine ambivalence, acknowledging both the benefits and risks of AI in a balanced manner. Responses such as “It has both advantages and disadvantages” or “AI can make us more creative. It can lead to over-reliance.” Fourth, passive avoidance. A distinct group expressed like “It's not something I can solve. I'll just use it as a tool and worry about problems later.”

## 6 Discussion

Regarding the first research question, the findings reveal that students' overall agreement with the five ethical principles leaning toward “neutral.” Furthermore, students showed a relatively higher agreement with the principles of “beneficence” and “autonomy” compared to the other three ethical principles, while their agreement with “non-maleficence,” “justice,” and “explicability” was relatively lower. A survey conducted by Yang et al. (2025) targeting university students found that college students had the lowest agreement with “justice” and the highest with “autonomy.” This suggests that there are differences in the perceptions of AI ethics among different age groups, highlighting that the introduction of AI ethics education in schools should not be viewed as the enforcement of rigid rules, but as a continuous and dynamic educational process based on students' performance, aimed at enriching their understanding of AI technology and promoting its responsible use. Experts have paid considerable attention to artificial intelligence ethics education, proposing multi-dimensional ethical frameworks to help students recognize the risks associated with AI technologies (Fjeld et al., 2020). When constructing ethical frameworks for artificial intelligence, education experts theoretically assume that all principles are equally important (Floridi and Cowls, 2022; Nguyen et al., 2022; Schiff et al., 2021). However, from the students' perspective, these ethical principles are treated differentially. This result shows the urgency and importance of current AI ethics education, while also reflecting the considerable work remains in its practical implementation.

For the second research question, there are several meaningful findings. First, the study revealed there is no significant differences in the level of agreement with AI ethical principles between students from urban and rural areas. It may suggest that, when strengthening AI education, resource allocation and policy focus should not assume an inherent urban-rural gap. Instead, greater focus should be placed on expanding access opportunities and improving the quality of experiences to ensure educational equity.

Second, students who have discussed AI-related topics with their parents demonstrate higher agreement with AI ethical principles compared to those who had not. This might indicate that home-school collaboration should be actively promoted, guiding families to become important spaces for AI ethics education. In fact, we designed a question to investigate whether students had attended AI lectures or training organized by their schools. The results showed that none of the students had participated in such activities. This reveals that if public schools do not these learning opportunities, children who cannot access relevant education from their families will be at a disadvantage (Dabbagh et al., 2025). These children often come from disadvantaged groups. Therefore, making AI a compulsory subject may be a necessary measure to prevent the further widening of the existing gaps in digital literacy and skills, known as the “digital divide” (Touretzky et al., 2019). UNESCO (2021) recently stated in its Recommendation on the Ethics of Artificial Intelligence that member states should provide adequate AI literacy education to the public at all levels in countries to empower people and reduce the digital divide and inequalities in digital access arising from the widespread use of AI systems. From an ethical perspective, implementing compulsory AI education also aligns with principles of fairness, justice, and equality.

Third, students with independent experience using generative AI tools show a higher agreement with AI ethical principles than those without such experience. This finding should be interpreted with caution because maybe students with higher digital literacy are more likely to explore AI and to hold more developed ethical views. Consequently, their higher-level agreement maybe not a direct outcome of use experience itself. However, this finding might also suggest that school education should go beyond theoretical instruction by designing safe and guided hands-on experiences with AI tools, allowing students to engage with AI ethics issues in practical contexts. Furthermore, higher agreement does not necessarily mean “better ethical understanding.” It may instead indicate greater exposure to ethical discourse. Students who discuss AI at home or use generative AI may have heard this phrase before or learned the “appropriate” responses to ethical questions without having internalized the underlying concepts.

Regarding the third research question, the themes summarizing students' rationales for agreeing, disagreeing with AI ethical principles, or hold a neutral stance uncover different attitudinal tendencies that may underlie students' agreement levels.

It is evident that middle school students' perceptions of AI ethical principles are not confined to academic integrity according to the rationales behind student's agreement with AI ethical principles. But some students show lower agreement with other ethical principles according to the rationales behind students' disagreement with AI ethical principles. Responses under themes like “human responsibility,” “avoidable,” and “can be solved” overlook the fact that AI technology is human-created and not value-neutral. Such an attitude might represent a dangerous misconception (Popenici, 2023). This may have been influenced by the broader societal atmosphere shaped by the media's predominantly positive coverage of AI development. Themes like “result-oriented” reflect students' tendency to prioritize AI utility over abstract ethics. Against the backdrop of the widespread use of generative AI as a learning tool to enhance student learning outcomes in Chinese middle schools in highly competitive environments, the outcome-oriented characteristic of educational assessment might lead students to perceive AI merely as an instrumental tool for achieving their goals.

Our qualitative analysis of neutral responses has identified four key themes. The theme of cognitive limitations suggests a primary reason for students' neutral stance toward AI ethics. This directly reflects the genuine cognitive starting point of adolescents when engaging with highly abstract and emerging issues. The theme of context-dependence illustrates that their ethical reasoning is highly specific and conditional, as they believe right or wrong depends on the purpose, context, and the actors involved. This is developmentally appropriate, pragmatic, or context-sensitive forms of ethical reasoning. The theme of contradiction and trade-offs indicates that students are capable of recognizing the multiple and often conflicting values introduced by AI technology and engage in internal deliberation, yet they find it difficult to arrive at definitive answers. This is aligned with conclusions of Li et al. (2025). They suggested that middle school students‘ understanding of AI ethics issues is constrained by their developmental level of abstract thinking. At the initial stage, a neutral stance reflects a normal manifestation of their stage of cognitive development. Their neutral stance often stems from unfamiliarity with concepts and difficulty in judgment. These findings indicate that ethical reasoning in adolescence is more nuanced than simple agreement/disagreement. Instead, they suggest that neutral responses often signal the most cognitively engaged stance. One characterized by genuine grappling with complexity, contextual nuance, and the limits of one's understanding. Therefore, in educational interventions, the primary task should perhaps not be to change the “neutral” stance, but rather to build scaffolding, provide relevant cases, and help these students transform their internal vague perceptions into ethical concepts. The theme of passive avoidance reveals that some middle school students are not ignorant of ethical issues but rather exhibit a form of resigned compliance based on pessimistic expectations.

The findings of this study provide guidance for designing age-appropriate AI ethics education in contexts with similar technological and educational resources to Eastern China. Educators can utilize these rationales as teaching examples and discussion starting points, helping to ground AI ethics education in the lived experiences and perspectives of adolescents. This approach can make lessons more relevant and engaging, thereby enhancing the effectiveness of AI ethics education. Below is a teaching strategy example. This strategy named “A Scenario-Based Learning Activity,” which aims to addresses students' tendency to prioritize AI utility over abstract ethics.

(a) Scenario Introduction (5 mins). Present a real-life scenario relevant to students' daily AI use. For example, “Your English homework app uses generative AI to solve problems, but it collects your personal information without permission. The app says this helps improve its accuracy, but you worry about your privacy. What would you do?”

(b)Group Discussion (15 mins). Divide students into groups to discuss two questions: (1) What are the benefits of this AI app for you? (2) What ethical problems might it cause? Guide students to list practical benefits and ethical concerns based on their own experiences.

(c)Ethical Principle Connection (15 mins). Introduce the ethical principle of “privacy” through concrete examples (e.g., your personal information is like your diary—others should not take it without your consent) instead of abstract definitions. Link the scenario to the principle: The app's data collection violates privacy (non-maleficence) because it uses your information without your agreement, even if it makes the app better.

(d)Decision-Making Practice (10 mins). Ask students to vote on three options: (1) Continue using the app and ignore privacy; (2) Stop using the app to protect privacy; (3) Ask the app developer to stop collecting data. After voting, have students explain their choices, emphasizing that there is no “perfect” answer but that ethical thinking requires balancing utility and responsibility.

## 7 Conclusion

This research explored the current state of middle school students' perceptions of AI ethics. The findings offer empirical evidence to support the design of tailored ethics curricula in primary and secondary schools, while also contributing to the global discourse on adolescents' understanding of AI ethics.

However, this research has the following limitations. First, the representativeness and scope of the sample are limited. Although the sample included both urban and rural schools, it was drawn from a single province and did not encompass regions with widely varying levels of economic development and educational resources. Moreover, the sample is limited by its geographical and cultural homogeneity, as the sample was exclusively drawn from middle school students in China. They may not be generalizable to students from different cultural backgrounds. Future research should include participants from diverse cultural and economic regions to enhance the generalizability of the findings. Second, the research instrument was simplified. Although this study was designed based on an existing AI ethics framework, primarily investigating students' agreement with five ethical principles and their rationales, it did not follow psychometric standard procedures to develop a scale. The findings of this study are best characterized as exploratory and hypothesis-generating rather than confirmatory. The patterns observed do not constitute definitive tests of theoretical relationships but rather serve to identify promising avenues for future research. Future scholars could develop an AI ethics scale for middle school students to strengthen the scientific robustness and accuracy of the measurement.

## Data Availability

The datasets presented in this article are not readily available due to containing information that could compromise the privacy of research participants. Requests to access the datasets should be directed to: Yanyan Zhang, yanerfive@gmail.com.
